# COMPARISON OF THE USE OF THE SURAL FLAP FOR THE MEDIAL, LATERAL, AND CENTRAL REGIONS OF THE ANKLE

**DOI:** 10.1590/1413-785220263403e299958

**Published:** 2026-06-12

**Authors:** Erick Yoshio Wataya, Jacqueline Alves Hosokawa, Krissia Caroline Soares Franco, Luiz Sorrenti, Luciano Ruiz Torres, João Carlos Nakamoto, Teng Hsiang Hei, Marcelo Rosa de Rezende, Rames Mattar

**Affiliations:** 1 Universidade de Sao Paulo, Faculdade de Medicina, Hospital das Clinicas, Instituto de Ortopedia e Traumatologia (HC-FMUSP), Sao Paulo, SP, Brazil.

**Keywords:** Microsurgery, Surgical Flaps, Risk Factors, Microcirurgia, Retalhos Cirúrgicos, Fatores de Risco

## Abstract

**Objective:**

To evaluate the occurrence of partial or total loss of the reverse-flow sural flap used for coverage failures in the lateral, medial, and central regions of the ankle, as well as factors related to flap loss.

**Methods:**

A retrospective cohort study was conducted including data from 32 patients who underwent surgery with reverse sural flaps between February 2012 and September 2023 at the Institute of Orthopedics and Traumatology, HC/FMUSP.

**Results:**

In the group of patients requiring coverage in the medial region, 40% had partial flap loss and 30% suffered total loss. In the lateral region, 20% experienced partial loss and 13.3% total loss. In the central area, 57.14% had partial coverage loss, with no cases of total loss.

**Conclusion:**

The reverse sural flap proved to be favorable for coverage of defects in the lateral region of the ankle, but showed higher rates of partial or total loss when used for coverage of defects in the medial region of the ankle. A negative effect was observed in the presence of patient comorbidities, in trauma-related cases, and in the early approach to flap construction. ***Level of Evidence II; Retrospective cohort study.***

## RESUMO

**Objetivo:**

Avaliar a ocorrência de perda parcial ou total do retalho sural de fluxo reverso utilizado nas falhas de cobertura na região lateral, medial e central do tornozelo, assim como fatores relacionados à perda do retalho.

**Métodos:**

Foi realizado um estudo de coorte retrospectiva incluindo os dados de 32 pacientes submetidos à cirurgia com retalhos surais reversos entre fevereiro de 2012 a setembro de 2023 no Instituto de Ortopedia e Traumatologia do HC/FMUSP.

**Resultados:**

No grupo de pacientes com necessidade de cobertura na região medial, 40% tiveram perda parcial do retalho e 30% sofreram perda total. Na região lateral, foram 20% de perdas parciais e 13,3% de perdas totais. E na área medial, 57,14% tiveram perda parcial da cobertura, sem nenhum caso de perda total.

**Conclusão:**

O retalho sural reverso se mostrou favorável para a cobertura de falhas na região lateral no tornozelo e mostrou maiores taxas de perdas parciais ou totais quando foi utilizado para a cobertura das falhas na região medial do tornozelo. Houve um efeito negativo na presença de comorbidades do paciente, nos casos causados por trauma e na abordagem precoce da confecção do retalho. ***Nível de Evidência II; Estudo de coorte retrospectiva.***

## INTRODUCTION

The choice of the best option for skin coverage in the distal leg and hindfoot is a difficult decision for surgeons. The scarcity of local flap tissues and the deficit of adequate vascularization, as seen in cases of severe trauma or arterial diseases (common causes of soft tissue injuries), are some of the factors that create difficulties in treating tissue loss in this region.^1^

Microsurgical flaps are alternatives for these cases, but they require adequate infrastructure and a skilled team for their execution, which is not available in the vast majority of hospital centers.

Donski and Fogdestam^2^ described the distal-based sural fasciocutaneous flap in 1983, and Masquelet et al.^3^ popularized the reverse sural fasciocutaneous flap in 1992, thus developing an excellent option for local flaps to cover areas in the ankle region.

**Figure 1:**
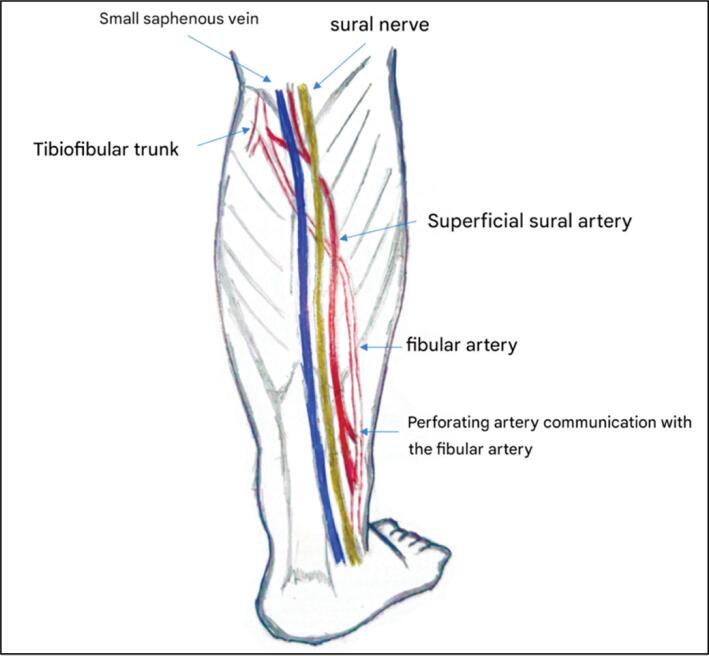
Perforating communicating branches between the superficial sural artery and the fibular artery.

The reverse sural flap is vascularized by the communicating and perforating branches of the fibular artery, which originate 5 to 6 cm cranially to the lateral malleolus (Figure 1), and due to its lack of technical complexity in execution, it shows good functional and aesthetic results.^4^

The flap depends on a retrograde arterial blood supply mainly from septocutaneous perforators of the fibular artery.^4^ It is also supplied by perforators from the posterior tibial artery, venous perforators associated with the small saphenous vein, and neurocutaneous perforators accompanying the sural artery.^4^

**Figure 2:**
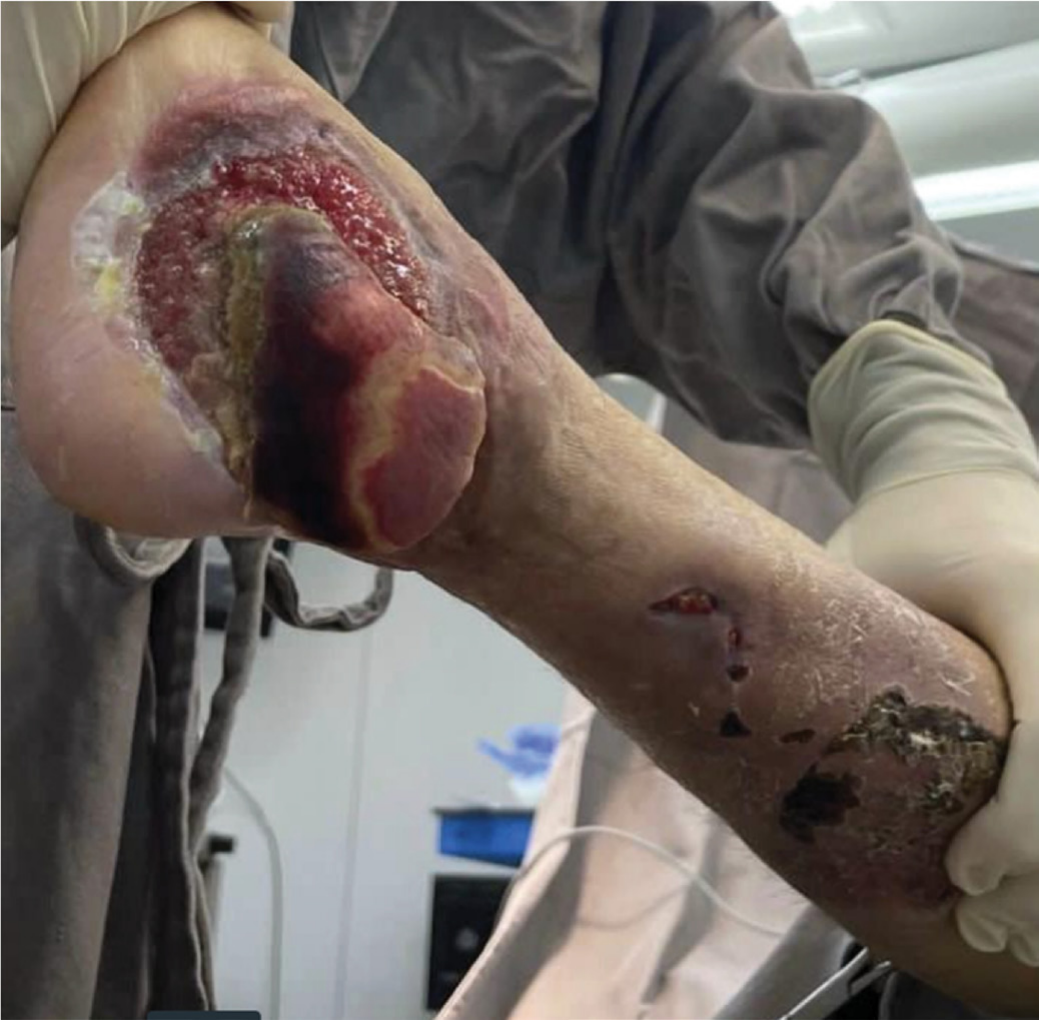
Case of total loss of the sural flap for coverage of a defect in the lateral region.

However, even with the ease of execution and the relatively constant anatomy of its pedicle, there are cases where partial or total loss of the flap occurs (Figure 2), which may be related to several factors such as: size of the skin defect, comorbidities, age, mechanism of trauma, time elapsed since the injury, among other factors.^1,5–8^

**Figure 3:**
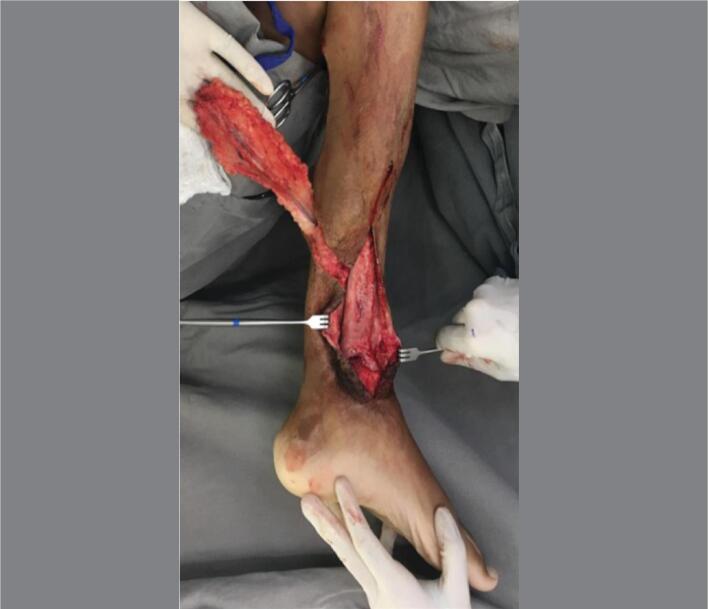
Medial skin failure at the ankle.

**Figure 4:**
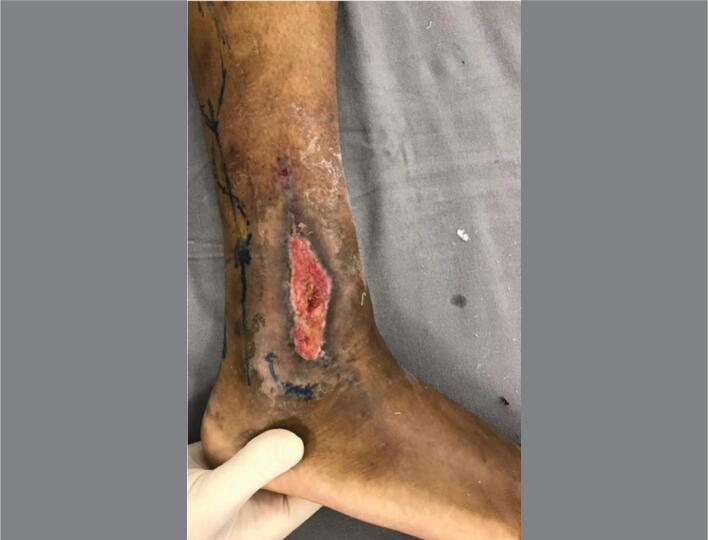
Lateral skin failure at the ankle.

**Figure 5:**
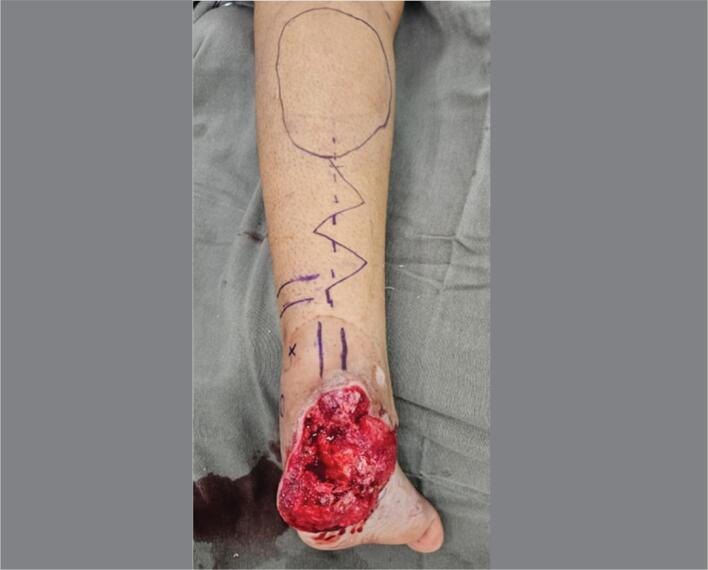
Central skin failure at the ankle.

Furthermore, it is still unknown in which region of the ankle – medial, lateral, or central (Figures 3, 4, and 5) – the highest success rate occurs, with a lower risk of complications from the reverse sural flap.^2–4^

Thus, in light of these variables that may influence the success rate of the reverse flow sural flap in different regions of the ankle, we decided to conduct this comparative study.

The primary objective of this study is to evaluate the occurrence of flap loss (partial or total) in coverage defects in the lateral, medial, and central regions of the ankle, and the secondary objective is to assess factors related to flap loss such as: presence of comorbidities, need for postoperative reintervention, etiological factor of skin loss, and the timing of when the patient underwent surgery (immediately after the initial cause, or later).

## METHODOLOGY

### Retrospective cohort study

Thirty-two patients who underwent surgery with reverse sural flaps from February 2012 to September 2023 at the Institute of Orthopedics and Traumatology of the Hospital das Clínicas of the Faculty of Medicine of the University of São Paulo were included. The study was approved by the Research Ethics Committee under number 64645122.2.0000.0068. All patients included in the study signed the informed consent form.

#### Inclusion Criteria

- Skin defects in the distal region of the leg/ankle - between the midfoot and the middle third of the leg.

#### Exclusion Criteria

- Circumferential defects in the ankle region.

#### Evaluation Criteria

- Location of the skin coverage defect (lateral, medial, or central region of the ankle);
- Surgical outcome (success, partial loss, or total loss of the flap);
- Mechanism of trauma or injury (trauma, infection, dehiscence of previous surgical wound);
- Need for postoperative surgical reintervention;
- Presence of comorbidities: Diabetes Mellitus (DM), smoking (SMK), peripheral vascular disease (PVD);
- Time elapsed until surgery: acute period (up to 48 hours after the initial cause) or later (after 48 hours from the initial cause).
  The data were analyzed by descriptive statistical analysis and presented in absolute and relative frequencies, and compared using the chi-square test with its association verified through relative risk. All analyses were performed using SPSS 24.0 software, with a significance level set at 95%.

## RESULTS

Of the 32 reverse sural flaps included in the study: ten cases were for coverage of the medial ankle region, fifteen cases for the lateral region, and seven for failures in the central ankle region.

In the group of patients requiring coverage in the medial region, 40% had partial flap loss and 30% suffered total loss. In the lateral region, there were 20% of partial losses and 13.3% of total losses. In the medial area, 57.14% experienced partial loss of coverage, with no cases of total loss (Table 1).

**Table 1:** Cases of flaps for medial coverage failures.

	Flap loss			
	Yes (n = 7)	No (n = 3)		
			RR (IC 95%)	Total (n = 10)
**Comorbidities**				
Yes	2 (20.0%)	2 (20.0%)	5.00 (0.27 – 91.52)	4 (40.0%)
No	5 (50.0%)	1 (10.0%)	0.20 (0.01 – 3.66)	6 (60.0%)
**Causes of coverage failure**				
Open fracture	4 (40.0%)	0	p 0.52	4 (40.0%)
Infection	1 (10.0%)	3 (30.0%)*		4 (40.0%)
Wound dehiscence	2 (20.0%)	0		2 (20.0%)
**Reintervention**				
Yes	6 (60.0%)	0		6 (60.0%)
No	1 (10.0%)	3 (30.0%)*	0.25 (0.04 – 1.36)	4 (40.0%)
**Chronicity**				
Acute	5 (50.0%)	0		5 (50.0%)
Chronic	2 (20.0%)	3 (30.0%)*	2.50 (0.85 – 7.13)	5 (50.0%)

RR: relative risk; 95% CI: 95% confidence interval;

* significant for the chi-square test; p < 0.05.

The most common causal indications among all patients were: open fracture (43.75%), infection (37.5%), and dehiscence of previous wounds (18.25%).

Regarding the mechanism of trauma or etiology of the injury in cases of failures in the medial region, 4 cases of open fracture, 1 case of infection, and 2 cases due to dehiscence of previous wounds experienced some degree of loss –partial or total – of the flap (Table 1).

On the lateral side of the leg, 2 skin failures caused by open fracture, 2 caused by infection, and 1 case caused by dehiscence experienced some degree of necrosis (Table 2).

**Table 2:** Cases of flaps for lateral coverage failures.

	Flap loss			
	Yes (n = 5)	No (n = 10)		
			RR^1^ (95% CI)	Total (n = 15)
**Comorbidities**				
Yes	3 (20.0%)	5 (33.3%)	1.14 (0.56 – 2.33)	8 (53.3%)
No	2 (13.3%)	5 (33.3%)	0.76 (0.17 – 3.32)	7 (46.7%)
**Cause of coverage failure**				
Open fracture	2 (13.3%)	3 (20.0%)		5 (33.3%)
Infection	2 (13.3%)	6 (40.0%)		8 (53.3%)
Wound dehiscence	1 (6.7%)	1 (6.7%)		2 (13.3%)
**Reintervention**				
Yes	4 (26.7%)	0*		4 (26.7%)
No	1 (6.7%)	10 (66.7%)*	0.09 (0.01 – 0.58)	11 (73.3%)
**Chronicity**				
Acute	4 (26.7%)	4 (26.7%)	3.50 (0.50 – 24.41)	8 (53.3%)
Chronic	1 (6.7%)	6 (40.0%)	0.58 (0.27 – 1.24)	7 (46.7%)

RR: relative risk; 95% CI: 95% confidence interval;

* significant for the chi-square test; p < 0.05.

In the central region, 3 cases secondary to open fracture and 1 case of dehiscence of the surgical wound experienced flap loss (Table 3). However, in all these cases, the p-value was not statistically significant.

**Table 3:** Cases of flaps for central coverage failures.

	Flap loss			
	Yes (n = 4)	No (n = 3)		
			RR (95% CI)	Total (n = 7)
**Comorbidities**				
Yes	4 (57.1%)	0*		4 (57.1%)
No	0	3 (42.9%)*		3 (42.9%)
**Cause of coverage failure**	
Open fracture	3 (42.9%)	2 (28.6%)	1.20 (0.25 – 5.70)	5 (71.4%)
Wound dehiscence	1 (%)	1 (%)	0.80 (0.13 – 4.61)	2 (28.6%)
**Reintervention**	
Yes	4 (57.1%)	0		4 (57.1%)
No	0	3 (42.9%)		3 (42.9%)
**Chronicity**	
Acute	4 (57.1%)	3 (42.9%)		7 (100.0%)
Chronic	0	0		0

RR: relative risk; 95% CI: 95% confidence interval;

* significant for the chi-square test; p < 0.05.

Of the total patients with failure in the medial region, we had 70% with some type of flap loss, both partial and total. Of these cases, 40% did not require any reintervention procedure, with a 75% lower risk of experiencing any flap loss compared to those who did not need reintervention (RR: 0.25). Additionally, 40% of the patients had some comorbidity, with a risk of experiencing flap losses 5 times greater than patients without comorbidities (RR: 5.00) (Table 1).

Comparatively, in the lateral region, we noted a 33.3% loss rate with 73.3% of patients not undergoing reintervention and a 91% lower risk of flap loss compared to those who were readdressed (RR: 0.09). Additionally, 53.3% had some comorbidity, presenting a 1.14 times greater chance of loss (RR: 1.14) (Table 2).

In relation to patients operated on to cover failures in the central region, 57.1% of this total underwent reintervention, resulting in loss in all cases, and all who had some comorbidity experienced some flap loss (Table 3).

Patients operated on for failures in the medial portion during the chronic phase, that is, in a period following the trauma mechanism, had 2.5 times higher chances of losing the flap compared to those operated on in the acute phase (RR: 2.5) (Table 1). In the lateral region, 53.3% were operated on acutely and had a 3.5 times greater chance of loss compared to those operated on in the chronic phase (RR: 3.50) (Table 2). All patients with skin failure in the central region of the leg were operated on in the acute phase (Table 3).

## DISCUSSION

Acute or chronic skin coverage losses in the distal region of the tibia or ankle have always posed a challenge for the surgeon in deciding the best indicated treatment. The coverage with free flaps is always an option; however, some centers do not offer adequate hospital structure, qualified medical staff, and nursing care for the execution of microsurgery; thus, the reverse sural flap, a locoregional flap, is an effective alternative in the treatment of these injuries.

The performance of the reverse sural flap does not require advanced microsurgical techniques, highly specialized teams, or high-complexity surgical centers compared to free flaps. The procedure requires a relatively short operative time and does not sacrifice any important artery. Additionally, it is capable of covering a wide area of skin failure. It is recommended for many soft tissue defects of the distal third of the leg, ankle, and dorsum of the foot, being capable of covering lateral, medial, or central regions of the lower limb.^8^

The complication rates after reconstruction with the reverse sural flap vary widely in the literature. Some authors point to worse outcomes related to factors such as: timing of surgery, comorbidities, and trauma mechanism.

In our study, of the 32 cases operated on, we obtained a total flap loss rate of around 15.6%, slightly above the average of other studies, which reported rates of 3% to 10%.^1,9–11^ The partial flap loss rate was also high, at approximately 34.4%, above the values reported even for free flaps of the lower limb.^1,9–11^

In teaching hospitals and referral centers for complex and emergency cases, the volume of surgeries is large, and patients often require multidisciplinary follow-up with a clinical team, trauma surgery, and microsurgery. As a result, some more severe cases may not be treatable for skin failure in the acute phase, and these cases often present other associated injuries such as fractures, nerve injuries, and vascular injuries. Moreover, since it is a teaching hospital, not all surgeries were performed by the same attending surgeon, which may influence the technical aspect of the procedure. However, all cases were operated on by a team of qualified microsurgeons with technical familiarity for performing the sural flap.

The results of this study differ from those demonstrated in the systematic and analytical review conducted by de Blacam et al.^10^ In this study, there was an overall complication rate of 26.4%, and total flap necrosis occurred in 3.2% of patients, mainly associated with DM, PVD and SMK.

Follmar et al.^4^ in their systematic review and meta-analysis of 2007 analyzed the results of the reverse sural flap, and 82% of cases healed without complications, with total flap loss in 3.3% and partial flap necrosis in 11% of cases. These loss rates were also associated with DM and PVD in up to 60%.

In our study, these comorbidities reported in the literature also appear to correlate with a higher loss rate, due to vascular diseases and other comorbidities, such as DM, which create unfavorable clinical conditions for flap integration.

Similar to what was found in our study, Johnson et al.^8^ found high rates of partial or even total flap loss in patients with comorbidities.^8^ However, Daar et al.^1^ (2020) found similar rates of complete loss compared to the healthy population; but higher rates of partial loss in the population with comorbidities associated with SMK.

The presence of comorbidity had a negative effect in our study for the three regions of the ankle studied, similar to other studies. However, due to the confidence interval, it was found that the results were not statistically significant, likely due to the small sample size of each group, leading us to believe that having comorbidity only tends to result in flap loss (p<0.05).

Regarding the etiological mechanism, skin failures secondary to trauma were more common, representing a rate of 43.75%, similar to the literature that presents rates ranging from 39.4 to 68.8%.^10,11^ This data reaffirms that, in hospitals considered reference centers, severe traumas represent a large part of the need for treatments for injuries with skin coverage failures, associated with other injuries. The highest expected flap loss rate according to location would be for the lateral regions, as the pivot point of the reverse sural flap is located laterally; for coverage failures in the lateral region, the pedicle needs to make a greater curvature for flap rotation, increasing the chances of kinking and compression of the pedicle, generating a higher risk of loss. However, in our study, the procedures designated for covering the medial region presented the highest total loss rate, at 70%. The reach of the flap to the medial region may be greater, which may leave the pedicle under tension, justifying the higher loss rate. We noted that, in coverage failures in the lateral region, those who required some reintervention procedure, such as surgical cleaning and repositioning of the flap, for example, had a lower tendency to experience any flap loss. In the medial region of the ankle, postoperative reintervention also showed a protective effect. One of the most common complications of reverse flow flaps is developing congestion, which can be identified with efficient and early monitoring, allowing for a rapid approach in cases of suspected distress. Our study demonstrated that early reintervention in cases of suspected ischemia or congestion in flaps for the medial and lateral regions can maintain flap viability.

The cases of failures in the medial portion operated on during the chronic phase had a 2.5 times higher chance of flap loss. However, all cases operated on during the acute phase experienced loss, which complicated the risk calculation of loss in this region.

In the lateral region, 43.7% operated in the chronic phase and showed 42% lower chances of loss. In the central region, all operated in the acute phase, with success rates of 57.1% for the flaps.

Patients in some cases were polytraumatized and often required multidisciplinary care, both clinical and surgical, necessitating multiple approaches until definitive treatment. This could hinder the creation of the flap in the early phase, requiring procedures for damage control in this initial phase.

According to Puneky et al.^12^, the reduction of time until definitive surgery helps decrease complication rates, with more satisfactory results in patients operated on within 72 hours of the injury. Cases operated on after 7 days of the initial trauma showed an increase in complications, with higher rates of infection and necrosis.^2—14^ Our study indicated a trend towards better outcomes in cases operated on later. The traumas that caused injuries with skin failure in the ankle region were severe, leading to extensive tissue loss. This may have led to a higher rate of necrosis in cases that were operated on during the acute phase, without adequate cleaning, debridement, and local damage control.

Our study has limitations regarding the small number of cases, especially when subdivided into the three major groups evaluated. However, we conducted a novel analysis comparing skin coverage failures in the medial, lateral, and central regions of the ankle, assessing their possible causes and rates of flap necrosis.

There was no statistical significance in the correlations, but we were able to indicate a trend towards better outcomes for failures in the lateral region of the ankle.

## CONCLUSION

The reverse sural flap showed a favorable trend for failures in the lateral region of the ankle and exhibited higher rates of partial or total losses in the medial region of the ankle.

A negative effect was observed in the presence of patient comorbidities, in cases caused by trauma, and in the early approach to flap construction.

## Data Availability

The authors confirm that all data supporting the findings of this study are fully available within the article.
